# Evaluation of changes in glycemic control and diabetic complications over time and factors associated with the progression of diabetic complications in Japanese patients with juvenile‐onset type 1 diabetes mellitus

**DOI:** 10.1111/1753-0407.13486

**Published:** 2023-10-18

**Authors:** Takafumi Masuda, Naoto Katakami, Hirotaka Watanabe, Naohiro Taya, Kazuyuki Miyashita, Mitsuyoshi Takahara, Ken Kato, Akio Kuroda, Munehide Matsuhisa, Iichiro Shimomura

**Affiliations:** ^1^ Department of Metabolic Medicine Osaka University Graduate School of Medicine Osaka Japan; ^2^ Department of Diabetes Care Medicine Osaka University Graduate School of Medicine Osaka Japan; ^3^ Diabetes Center, NHO Osaka National Hospital Osaka Japan; ^4^ Diabetes Therapeutics and Research Center Institute of Advance Medical Sciences, Tokushima University Tokushima Japan

**Keywords:** atherosclerosis, diabetic nephropathy, glycemic control, juvenile‐onset diabetes mellitus, type 1 diabetes mellitus

## Abstract

**Background:**

This study aimed to evaluate the changes in glycemic control and diabetic complications over time in Japanese patients with juvenile‐onset type 1 diabetes mellitus and to clarify the factors associated with the progression of diabetic complications.

**Methods:**

We tracked 129 patients with type 1 diabetes mellitus (21.8 ± 4.1 years old [mean ± SD] with a diabetes duration of 12.6 ± 5.7 years) for up to 19 years and analyzed data on glycated hemoglobin (HbA1c) and indicators related to the severity of diabetic complications (estimated glomerular filtration rate [eGFR], urinary albumin excretion rate [UAE], carotid intima‐media thickness [CIMT], and brachial‐ankle pulse wave velocity [baPWV]) using linear mixed model and decision tree analysis.

**Results:**

Although the HbA1c and UAE levels improved over time, the eGFR, CIMT, and baPWV worsened. Decision tree analysis showed that HbA1c and the glycoalbumin/HbA1c ratio for eGFR; HbA1c and systolic blood pressure for UAE; low‐density lipoprotein cholesterol/high‐density lipoprotein cholesterol ratio, glycoalbumin/HbA1c ratio, and body mass index (BMI) for CIMT; and HbA1c for baPWV were associated factors.

**Conclusions:**

In this retrospective observational study, glycemic control and albuminuria improved; however, renal function and arteriosclerosis worsened over time. HbA1c levels, glycemic excursion, and blood pressure are associated with nephropathy progression. HbA1c levels, glycemic excursion, lipid levels, and BMI are associated with the progression of atherosclerosis.

## Graphical abstract

**Highlights**

- Glycemic control and complications were examined in Japanese patients with type 1 diabetes mellitus.
- Glycated hemoglobin (HbA1c) and urinary albumin excretion rates improved over time.
- HbA1c, glycemic excursion, and blood pressure are related to diabetic nephropathy.
- HbA1c levels, glycemic excursion, lipid levels, and body mass index are associated with atherosclerosis.

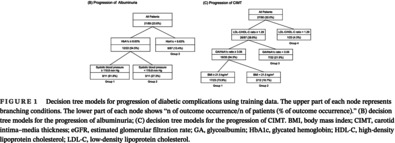

## 1 INTRODUCTION

The development of early microvascular complications during adolescence is not rare in patients with juvenile‐onset type 1 diabetes (T1D),^1^ and the development of microvascular complications leads to a decrease in the patient's quality of life. Macrovascular complications have been reported as the main cause of death in juvenile‐onset T1D.^2^, ^3^ Therefore, investigating changes in glycemic control and diabetic complications over time and the factors associated with the progression of diabetic complications in juvenile‐onset T1D is important for selecting appropriate treatment strategies, reducing the risk of diabetic complications, and improving long‐term outcomes.

Several studies have been conducted in Western countries on the changes in glycemic control and diabetic complications over time in juvenile‐onset T1D, and various factors associated with the progression of diabetic complications have been reported. Diabetes Patient History Documentation (DPV) and Western Australian Children's Diabetes Database registries in Germany and Australia, and the SWEET project (Better Control in Pediatric and Adolescent DiabeteS: Working to CrEate CEnTers of Reference), including 22 centers in Europe, Australia, and Canada, have shown improvements in glycated hemoglobin (HbA1c) over time in juvenile‐onset T1D.^4^, ^5^ Finnish and Australian cohort studies have also reported a decrease in incidences in microvascular and macrovascular complications over time in juvenile‐onset T1D.^6^, ^7^, ^8^ In addition, the DPV registry and SEARCH for Diabetes in Youth study have reported various factors associated with the progression of diabetic complications in juvenile‐onset T1D.^9^, ^10^, ^11^, ^12^

Conversely, in Japan, very few reports exist on changes in glycemic control and diabetic complications in patients with juvenile‐onset T1D. Reports are only on HbA1c in childhood‐onset T1D that improves over time and that the incidence of end‐stage renal disease decreases over time in patients with T1D with an onset of <30 years.^13^, ^14^ In addition, very few studies have reported factors associated with the progression of diabetic complications in Japanese patients with juvenile‐onset T1D.

Therefore, we collected data on the glycemic control and diabetic complications in Japanese patients with juvenile‐onset T1D. The purpose of our study was to analyze these data to clarify the changes in glycemic control and diabetic complications over time and the factors associated with the progression of diabetic complications in Japanese patients with juvenile‐onset T1D.

## 2 METHODS

### 2.1 Study population

This was a retrospective observational study using a data set obtained from a health check program for patients with T1D with an age at diagnosis of <20 years. This program is performed annually at Osaka University Hospital and Osaka Police Hospital. A total of 129 patients who participated in this program more than once between 2001 and 2019 were included in this study. All procedures were in accordance with the ethical standards of the responsible committee on human experimentation (institutional and national) and the Helsinki Declaration of 1964, as revised in 2013. This study was approved by the Ethics Committee of Osaka University Hospital, Japan (approval number: 14328‐9). Informed consent was obtained from all the patients to participate in the study. This study was also conducted according with Strengthening the Reporting of Observational Studies in Epidemiology guidelines.^15^

### 2.2 Clinical and biochemical measurements

Clinical data regarding age, sex, age at diagnosis, duration of diabetes, smoking, continuous subcutaneous insulin infusion (CSII), body mass index (BMI), blood pressure, statins, antihypertensives (angiotensin‐converting enzyme inhibitors, angiotensin receptor blockers, and calcium antagonists), and retinopathy were collected.

Biochemical data included HbA1c, glycoalbumin (GA), total cholesterol (TC), low‐density lipoprotein cholesterol (LDL‐C), triglyceride (TG), high‐density lipoprotein cholesterol (HDL‐C), LDL/HDL ratio, non‐HDL‐C, estimated glomerular filtration rate (eGFR), and urinary albumin excretion rate (UAE). All biochemical data were measured using standard laboratory protocols, and laboratory analyses were performed by SRL Inc. (Tokyo, Japan). Until 2011, HbA1c values were measured by latex agglutination turbidimetry using a set of calibrators assigned by the Japan Diabetes Society (JDS) (normal range 4.3%–5.8%). Therefore, HbA1c values were re‐evaluated as a National Glycohemoglobin Standardization Program equivalent value, calculated using the formula: HbA1c (%) = 1.02 × HbA1c (JDS) (%) + 0.25%.^16^ LDL‐C levels were calculated using the Friedewald formula: LDL‐C = TC − HDL‐C − TG/5. Non‐HDL‐C was calculated using the following formula: non‐HDL‐C = TC‐HDL‐C. The GA data were available from 2003.

Diabetic retinopathy was diagnosed by a diabetologist based on the findings of single‐field fundus photography based on the Davis classification. The diagnosis made by an ophthalmologist was confirmed via interviewing the patients. In this study, retinopathy was defined as existence of retinopathy more severe than or equal to simple diabetic retinopathy, according to the Davis classification.

### 2.3 Assessment of glycemic variability

Glucose profiles were assessed by measuring blood glucose seven times (before and after breakfast, lunch, and supper, and before bedtime). The Morbus value (M‐value)^17^ and the area under the curve (AUC) of glucose were calculated. These data were available from 2005.

Hypoglycemia was assessed through interviewing participants regarding the number of occurrences of severe hypoglycemia in the past 1 year. Severe hypoglycemia was defined as an event with severe cognitive impairment (including coma and convulsions) requiring external assistance from another person to correct the hypoglycemia.^18^ Hypoglycemic data were available from 2002.

The GA/HbA1c ratio was also used as a glycemic variability index. The GA/HbA1c ratio correlates with the mean amplitude of glucose excursion in T1D and is a marker of glycemic variability.^19^ GA/HbA1c ratio data were available from 2003.

### 2.4 Assessment of diabetic nephropathy

As indicators of diabetic nephropathy, we used eGFR and UAE, which are recommended screening for renal evaluation in the American Diabetes Association “Standards of Medical Care in Diabetes.”^20^ The eGFR was estimated using the eGFR equation for Japanese people, calculated using the following formula: eGFR = 194 × serum creatinine^−1.094^ × age^−0.287^ × 0.739 (if female).^21^ UAE was calculated as urinary albumin per day and urinary creatinine (Cre) per day, which were obtained through 24‐h urine collection.

### 2.5 Assessment of subclinical atherosclerosis

To assess subclinical atherosclerosis, we used carotid intima‐media thickness (CIMT) and brachial‐ankle pulse wave velocity (baPWV), indicators that have already been established as useful surrogates for atherosclerotic disease. CIMT and baPWV have been reported to be associated with cardiovascular events and mortality risk as indicators of atherosclerosis, and both reflect different aspects of atherosclerosis.^22^, ^23^, ^24^ IMT is a quantitative indicator of arterial wall thickening that reflects structural changes in the arteries, whereas baPWV is an indicator of vascular elasticity that reflects functional changes in the arteries.

CIMT was evaluated using B‐mode imaging ultrasonography with a high‐frequency linear probe (>7.5 MHz) (SSA‐340A and Aplio MX, TOSHIBA Medical Systems Inc., Tokyo, Japan). CIMT was measured based on the guidelines of the Japan Society of Ultrasonics.^25^ The CIMT was defined as the maximum thickening point, including the plaque of the common carotid artery, carotid bulb, and internal carotid artery in the longitudinal or transverse view. CIMT was measured on both sides of the carotid artery, and the larger part of either the left or right side was adopted as a representative value.

baPWV was evaluated using PWV/ankle‐brachial pressure index (Omron Colin, Tokyo, Japan) as previously reported.^24^, ^26^, ^27^ After the patient rested in the supine position for at least 5 min, blood pressure, and pulse volume waveforms of both the upper arm and ankle were measured. baPWV was calculated from the difference between the distance between two points estimated from the height and the time when the pulse waveform was propagated.^27^ The higher value of either the left or right baPWV was adopted as the representative value. baPWV data were available from 2005.

### 2.6 Statistical analysis

Missing values were not supplemented, and data at or below the lower detection limit were assigned a value equivalent to half the lower detection limit. Continuous variables were expressed as mean ± SD or median (first quartile point, third quartile point), and categorical variables as frequency (%), unless otherwise mentioned. Data that deviated greatly from normal distribution (TG and UAE) were log‐transformed. Normal distribution was visually confirmed using histograms and quantile‐quantile plots.

The data set used in this study consisted of repeated measurement data from different patients for each year. To evaluate changes in parameters, such as glycemic control and diabetic complications over time, we analyzed the data using a linear mixed model through the R package lmerTest (R Development Core Team, Vienna, Austria). Data were analyzed with the year of visit as a fixed effect and the patient as a random effect. One change in fixed effects, namely, the estimated change in each parameter per year, is denoted as “Estimate.”

To analyze the factors associated with the progression of diabetic nephropathy and arteriosclerosis, we performed a decision tree analysis. Decision tree analysis creates a tree‐structured prediction model by combining branching with explanatory variables. This analysis can handle nonlinear relationships and can flexibly treat interaction effects among covariates, and the results interpreted easily.^28^

To set the outcomes for the progression of diabetic nephropathy and arteriosclerosis, we calculated the annual changes in eGFR, Log UAE, CIMT, and baPWV for each patient using a linear regression model and divided the annual changes into quartile groups. For the eGFR, the occurrence of an outcome was defined as belonging to the lowest quartile. For the UAE, CIMT, and baPWV, the occurrence of the outcome was defined as belonging to the largest quartile.

Decision tree analysis was performed using the R package rpart (R Development Core Team), and the results were output using the default settings. For the objective variable, the occurrence of each outcome was set to 1 and nonoccurrence to 0. The explanatory variables included age, sex, age at diagnosis, duration of diabetes, smoking, retinopathy, and the mean parameters calculated using the trapezoidal rule method using data from the observation period (CSII use, BMI, HbA1c, GA/HbA1c ratio, LDL‐C, TG, HDL‐C, LDL‐C/HDL‐C ratio, non‐HDL‐C, statin use, systolic blood pressure [SBP], diastolic blood pressure, antihypertensive use, M‐value, AUC of glucose, severe hypoglycemia, eGFR, and UAE). For instance, in the case of HbA1c, mean HbA1c was calculated through the trapezoidal rule method using the following equation (assuming that the baseline HbA1c value is X_0_, the year of visit after the baseline is A, B, C,…, and the HbA1c value at that time is X_A_, X_B_, X_C_,…):

$$\text{Mean}\;\mathrm{HbA}1c=\begin{array}{l} 1/2\times (\left(X_{0}+X_{A}\right)/\left(A-0\right)\\ +\;\left(X_{A}+X_{B}\right)/\left(B-A\right)\\ +\;\left(X_{B}+X_{C}\right)/\left(C-A\right)+\ldots ) \end{array}$$

For smoking, retinopathy, CSII use, and use of statins and antihypertensives, yes was set to 1, and no was set to 0. Regarding eGFR, the values calculated using the trapezoidal rule method were classified into four groups and treated as categorical variables (1: eGFR ≥135 mL/min/1.73 m^2^, 2: 135 > eGFR ≥90 mL/min/1.73 m^2^, 3: 90 > eGFR ≥60 mL/min/1.73 m^2^, 4: eGFR <60 mL/min/1.73 m^2^). To create each prediction model, the data set was randomly assigned to two groups of training and test data at a ratio of 7:3. We then used training data to develop a predictive model for each outcome using the 10‐fold cross‐validation. Next, we verified each model using the test data. We calculated the accuracy, sensitivity, specificity, positive predictive value, and negative predictive value from the confusion matrix as the performance metrics.

All statistical analyses were performed using the R statistical software (version 4.2.1; R Development Core Team). Two‐sided *p* values of <.05 were considered statistically significant.

### 2.7 Sensitivity analysis

We examined how missing values affected outcomes using sensitivity analysis. We ran a sensitivity analysis for the linear mixed model using patients with observation periods of ≥2 and ≥10 points. The decision‐tree model's sensitivity analysis was repeated for patients without missing values.

## 3 RESULTS

### 3.1 Patient characteristics

A total of 129 patients with T1D were analyzed at 1403 observation points (data were missing for some evaluation items). The median follow‐up period was 9.0 years (interquartile range 4.0–12.0 years). Table 1 shows the baseline characteristics of all patients. A total of 47 males (36.4%) and 82 females (63.6%) were aged 21.8 ± 4.1 years (mean ± SD) with a diabetes duration of 12.6 ± 5.7 years, BMI of 22.4 ± 2.9 kg/m^2^, and HbA1c of 8.3 ± 1.6%. The CSII was used in 16 patients (12.8%). LDL‐C was 97.5 ± 23.9 mg/dL, and no patients were taking statins. SBP was 115.9 ± 12.5 mm Hg, diastolic blood pressure was 70.1 ± 10.8 mm Hg, and no patients were taking antihypertensives. Retinopathy was observed in 41 patients (32.0%). UAE (*n* = 128) was 6.0 (4.1–9.4) mg/g Cre (geometric mean [interquartile range]), and 8 patients (6.3%) had albuminuria (UAE ≥30 mg/g Cre). CIMT was 0.75 ± 0.11 mm, and baPWV (*n* = 39) was 1139.3 ± 174.8 cm/s.

**TABLE 1:** Characteristics at baseline of all patients (*n* = 129).

Parameters	
Age (years)	21.8 ± 4.1
Male (%)	47 (36.4%)
Age at diagnosis (years)	9.2 ± 5.0
Duration of diabetes (years)	12.6 ± 5.7
Smoking (%)	29 (22.5%)
CSII use (%)^a^	16 (12.8%)
BMI (kg/m^2^)	22.4 ± 2.9
HbA1c (%)	8.3 ± 1.6
GA (%)^b^	29.7 ± 9.9
GA/HbA1c ratio^b^	3.6 ± 1.2
LDL‐C (mg/dL)	97.5 ± 23.9
TG (mg/dL)	74.0 (56.0–108.0)
Log_10_TG (mg/dL)	1.9 ± 0.2
HDL‐C (mg/dL)	68.1 ± 12.9
LDL‐C/HDL‐C ratio	1.5 ± 0.5
Non‐HDL‐C (mg/dL)	116.9 ± 29.1
Statin (%)	0 (0%)
Systolic blood pressure (mm Hg)	115.9 ± 12.5
Diastolic blood pressure (mm Hg)	70.1 ± 10.8
Antihypertensives (%)	0 (0%)
M‐value^c^	136.9 ± 101.1
AUC of glucose (mg/dL h)^c^	1720.8 ± 496.7
Severe hypoglycemia (times/year)^d^	0.78 ± 3.8
Retinopathy^e^	41 (32.0%)
eGFR (mL/min/1.73 m^2^)	114.0 ± 19.9
UAE (mg/g Cre)^e^	6.0 (4.1–9.4)
Log_10_UAE (mg/g Cre)^e^	0.86 ± 0.40
Albuminuria (%)^e^	8 (6.3%)
CIMT (mm)	0.75 ± 0.11
baPWV (cm/s)^f^	1139.3 ± 174.8

*Note*: Categorical data are expressed as *n* (%), and continuous data as mean ± SD or geometric mean (interquartile range). Antihypertensive drugs include angiotensin‐converting enzyme (ACE) inhibitors, angiotensin receptor blockers, and calcium antagonists.

Abbreviations: AUC, area under the curve; baPWV, brachial–ankle pulse wave velocity; BMI, body mass index; CIMT, carotid intima‐media thickness; Cre, creatinine; CSII, continuous subcutaneous insulin infusion; eGFR, estimated glomerular filtration rate; GA, glycoalbumin; HbA1c, glycated hemoglobin; HDL‐C, high‐density lipoprotein cholesterol; LDL‐C, low‐density lipoprotein cholesterol; M‐value, Morbus value; TG, triglyceride; UAE, urinary albumin excretion rate.

^a^ *n* = 125.

^b^ *n* = 54.

^c^ *n* = 26.

^d^ *n* = 69.

^e^ *n* = 128.

^f^ *n* = 39.

### 3.2 Changes in each parameter over time

Table 2 shows the results of the changes in each parameter over time using a linear mixed model, using the year of visit as a fixed effect and the patient as the random effect. “Estimate” is the estimated amount of change in each parameter per year. The BMI, LDL‐C, HDL‐C, LDL‐C/HDL‐C ratio, non‐HDL‐C, sBP, CIMT, and baPWV increased significantly over time. Conversely, HbA1c level, GA level, GA/HbA1c ratio, M‐value, AUC of glucose level, eGFR, and Log UAE decreased significantly over time. These results remained significant even after adjusting for age and sex. Results of the sensitivity analyses were similar to those of the original analyses (Supplementary Tables S1 and S2).

**TABLE 2:** Annual change in each parameter evaluated using a linear mixed model (*n* = 129).

Parameters	Estimate	*p* value
BMI (kg/m^2^/year)	0.105	<.001
HbA1c (%/year)	−0.042	<.001
GA (%/year)^a^	−0.727	<.001
GA/HbA1c ratio (/year)^a^	−0.080	<.001
LDL‐C (mg/dL/year)	0.720	<.001
Log_10_TG (mg/dL/year)	−0.001	.329
HDL‐C (mg/dL/year)	0.131	.005
LDL‐C/HDL‐C ratio (/year)	0.009	<.001
Non‐HDL‐C (mg/dL/year)	0.736	<.001
Systolic blood pressure (mm Hg/year)	0.258	<.001
Diastolic blood pressure (mm Hg/year)	0.074	.054
M‐value (/year)^b^	−1.712	.012
AUC of glucose (mg/dL h/year)^b^	−6.689	.014
Severe hypoglycemia (times/year^2^)^a^	0.011	.335
eGFR (mL/min/1.73 m^2^/year)	−1.310	<.001
Log_10_UAE (mg/g Cre/year)	−0.011	<.001
CIMT (mm/year)	0.027	<.001
baPWV (cm/s/year)^c^	10.67	<.001

*Note*: “Estimate” is the estimated amount of change in each parameter per year.

Abbreviations: AUC, area under the curve; baPWV, brachial–ankle pulse wave velocity; BMI, body mass index; CIMT, carotid intima–media thickness; Cre, creatinine; eGFR, estimated glomerular filtration rate; GA, glycoalbumin; HbA1c, glycated hemoglobin; HDL‐C, high‐density lipoprotein cholesterol; LDL‐C, low‐density lipoprotein cholesterol; M‐value, Morbus value; TG, triglyceride; UAE, urinary albumin excretion rate.

^a^ *n* = 128.

^b^ *n* = 117.

^c^ *n* = 125.

Furthermore, we analyzed the association between CSII use and HbA1c level, GA/HbA1c ratio, and M‐values using a linear mixed model. CSII use was associated with a reduction in HbA1c level, GA/HbA1c ratio, and M‐values (Supplementary Table S3).

### 3.3 Factors associated with progression of diabetic nephropathy and atherosclerosis

**FIGURE 1:**
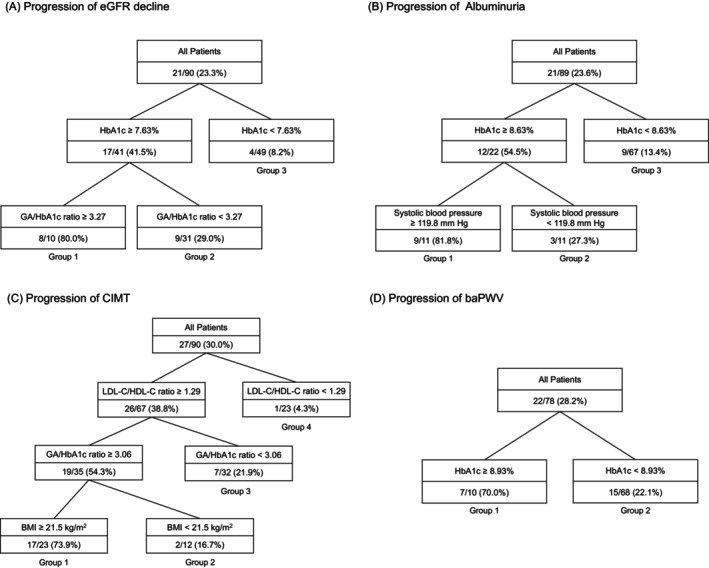
Decision tree models for progression of diabetic complications using training data. The upper part of each node represents branching conditions. The lower part of each node shows “n of outcome occurrence/n of patients (% of outcome occurrence).” (A) Decision tree models for the progression of eGFR decline; (B) decision tree models for the progression of albuminuria; (C) decision tree models for the progression of CIMT; and (D) decision tree models for the progression of baPWV. baPWV, brachial–ankle pulse wave velocity; BMI, body mass index; CIMT, carotid intima–media thickness; eGFR, estimated glomerular filtration rate; GA, glycoalbumin; HbA1c, glycated hemoglobin; HDL‐C, high‐density lipoprotein cholesterol; LDL‐C, low‐density lipoprotein cholesterol.

Annual changes in eGFR, LogUAE, CIMT, and baPWV calculated using the linear regression model were −1.071 (−3.616–[−0.058]) mL/min/1.73 m^2^/year for eGFR, −0.125 (−0.364–0.419) mg/g Cre/year for LogUAE, 0.021 (0.010–0.035) mm/year for CIMT, and 11.13 (4.21–22.19) cm/s/year for baPWV (geometric mean [interquartile range], respectively). Figure 1A–D shows the results of a prediction model created from training data using decision tree analysis for factors associated with the progression of diabetic nephropathy and arteriosclerosis.

Figure 1A is the prediction model for the progression of eGFR decline. Outcomes were observed in 21 patients (23.3%) in the training data set. In 10 patients with HbA1c ≥7.63% and GA/HbA1c ratio ≥3.27 (Group 1), the outcome occurred at a high rate of 80.0% (8 of 10 patients). In 31 patients with HbA1c ≥7.63% and GA/HbA1c ratio <3.27 (Group 2), the outcome occurred in 9 patients (29.0%). In 49 patients with HbA1c <7.63% (Group 3), the outcome occurred in 9 patients (8.2%). When this model was verified using the test data (*n* = 39), the accuracy, sensitivity, specificity, positive predictive value, and negative predictive value were 0.77, 0.45, 0.89, 0.63, and 0.81, respectively.

Figure 1B is the prediction model for UAE progression. Outcomes were observed in 21 patients (23.6%) in the training dataset. In 11 patients with HbA1c ≥8.63% and sBP ≥119.8 mm Hg (Group 1), the outcome occurred at a high rate of 81.8% (nine of 11 patients). In 11 patients with HbA1c ≥8.63% and SBP <119.8 mm Hg (Group 2), the outcome occurred in three patients (27.3%). In 67 patients with HbA1c <8.63% (Group 3), the outcome occurred in 9 patients (13.4%). When this model was verified using the test data (*n* = 39), the accuracy, sensitivity, specificity, positive predictive value, and negative predictive value were 0.74, 0.09, 1.00, 1.00, and 0.74, respectively.

Figure 1C is the prediction model for CIMT progression. Outcomes were observed in 27 patients (30.0%) in the training data set. In 23 patients with LDL‐C/HDL‐C ratio ≥1.29 and GA/HbA1c ratio ≥3.06 and BMI ≥21.5 kg/m^2^ (Group 1), the outcome occurred at a high rate of 73.9% (17 of 23 patients). In 12 patients with LDL‐C/HDL‐C ratio ≥1.29 and GA/HbA1c ratio ≥3.06 and BMI <21.5 kg/m^2^ (Group 2), the outcome occurred in 2 patients (16.7%). In 32 patients with LDL‐C/HDL‐C ratio ≥1.29 and GA/HbA1c ratio <3.06 (Group 3), the outcome occurred in 7 patients (21.9%). In 23 patients with an LDL‐C/HDL‐C ratio <1.29 (Group 4), the outcome occurred in 1 patient (4.3%). When this model was verified using the test data (*n* = 39), the accuracy, sensitivity, specificity, positive predictive value, and negative predictive value were 0.72, 0.20, 0.79, 0.13, and 0.87, respectively.

Figure 1D is the prediction model for baPWV progression. Outcomes were observed in 22 patients (28.2%) in the training data set. In 10 patients with HbA1c ≥8.93% (Group 1), the outcome occurred in seven patients (70.0%). In 68 patients with HbA1c <8.93% (Group 2), the outcome was observed in 15 patients (22.1%). When this model was verified using the test data (*n* = 34), the accuracy, sensitivity, specificity, positive predictive value, and negative predictive value were 0.85, 0.50, 0.93, 0.60, and 0.90, respectively.

The results of the sensitivity analyses revealed almost the same associated factors as those in the original analysis (Supplementary Figure S1).

## 4 DISCUSSION

### 4.1 Changes in glycemic control and clinical parameters in juvenile‐onset T1D over time

The change in HbA1c over time for all 129 patients analyzed using a linear mixed model was −0.042%/year (Table 2), and HbA1c decreased over the 19 years from 2001 to 2019. GA also decreased over time (Table 2). These results are consistent with those of previous studies that reported improvements in glycemic control over time in patients with T1D.^4^, ^5^, ^29^ In addition, a previous study reported that increased CSII use contributed to improved glycemic control over time.^4^ In our study, 16 of 125 patients (12.8%, 4 with missing data) used CSII at baseline, and CSII use increased to 54 of 128 patients (42.2%, one with missing data) at the end of the observation period. In a previous study, we reported an improvement in glycemic control in 35 patients who participated in our health check program and whose treatment had been changed from multiple daily injections (MDI) to CSII during the observation period.^30^ In this study, CSII use was associated with the reduction in HbA1c levels (Supplementary Table S3). Therefore, one of the reasons for the improvements in glycemic control over time in this population is thought to be advances in treatment technology, such as an increase in CSII use.

As indicators of glycemic excursion, the GA/HbA1c ratio and M‐values decreased over time. CSII use was also associated with the reduction in GA/HbA1c ratio and M‐values (Supplementary Table S3). Therefore, one of the reasons for the improvements in the indicators of glycemic excursion is thought to be the increase in CSII use.

In this study, BMI increased over time. A DPV registry in Germany reported that BMI increased from 25.0 to 26.2 kg/m^2^ from 1999 to 2018,^31^ and this result is consistent with our study. In our study, the LDL‐C and SBP levels increased over time. LDL‐C and SBP were correlated with BMI (data not shown) suggesting that weight gain contributed to increases in LDL‐C and SBP levels.

### 4.2 Factors associated with the progression of diabetic nephropathy and atherosclerosis in juvenile‐onset T1D

Decision tree analysis showed that the progression of eGFR decline occurred more frequently in patients with high HbA1c levels and GA/HbA1c ratios (Figure 1A). The annual change in eGFR in the quartile group with the lowest annual change, which was set as the outcome of this study, was −3.616 mL/min/1.73 m^2^/year or less. An annual eGFR change of −3 mL/min/1.73 m^2^/year or less is generally regarded as a rapid eGFR decline, and early decline in eGFR is a risk factor for end‐stage renal disease in T1D.^32^ Age, duration of diabetes, HbA1c level, UAE, and hyperfiltration have been reported as factors associated with a rapid eGFR decline in juvenile‐onset T1D.^33^, ^34^, ^35^ Conversely, factors associated with rapid eGFR decline in Japanese patients with juvenile‐onset T1D have not been reported. This study is the first to examine this point and to reveal that the GA/HbA1c ratio is associated with a rapid eGFR decline in juvenile‐onset T1D. GA/HbA1c has been reported as a marker of glycemic excursions in T1D,^19^ and endothelial cell damage and oxidative stress induced by glucose excursions leading to a decline in eGFR are possible.

Decision tree analysis showed that UAE progression of UAE was more frequent in patients with both high HbA1c and high SBP (Figure 1B). Several longitudinal studies on juvenile‐onset T1D conducted in Western countries have reported risk factors for the progression of UAE, including age; male sex; duration of diabetes; smoking; presence of retinopathy; HbA1c, LDL‐C, and TG levels; and blood pressure.^36^, ^37^, ^38^ Two longitudinal studies of Japanese patients with juvenile‐onset T1D investigating the factors associated with the progression of albuminuria and the introduction of renal replacement therapy reported an association between age at diagnosis and HbA1c.^39^, ^40^ Our findings are consistent with those of previous studies. Notably, the Diabetes Control and Complications Trial (DCCT), a randomized controlled trial of T1D, and a subsequent observational study, the Epidemiology of Diabetes Interventions and Complications (EDIC), reported that low blood pressure (120/70 mm Hg or less) reduced the risk of albuminuria regardless of treatment during DCCT.^41^ Our criteria of less than 120 mm Hg in sBP was consistent with those of the DCCT/EDIC study.

Decision tree analysis showed that CIMT progression was more frequent in patients with a high LDL‐C/HDL‐C ratio, GA/HbA1c ratio, and BMI (Figure 1C). The association of LDL‐C/HDL‐C ratio and BMI with CIMT has already been shown in the DCCT/EDIC and SEARCH studies,^9^, ^42^ and our study confirmed these associations in Japanese patients with juvenile‐onset T1D. The association between GA/HbA1c ratio and CIMT has been reported in patients with type 2 diabetes mellitus.^43^ Conversely, in T1D, our study is the first to report this association. Although glucose excursions are widely known to promote atherosclerosis, the GA/HbA1c ratio has also been shown to be associated with the progression of CIMT not only in patients with type 2 diabetes mellitus but also in those with T1D.

Decision tree analysis showed that baPWV progression occurred more frequently in patients with high HbA1c (Figure 1D). Very few longitudinal studies have reported the factors associated with PWV progression in juvenile‐onset T1D, and this study is the first to report on Japanese patients with juvenile‐onset T1D. Two of the SEARCH studies reported that PWV progression was associated with waist circumference, LDL‐C, HbA1c, age, duration of diabetes, smoking status, insulin sensitivity, and mean arterial blood pressure.^10^, ^11^ In our study, we did not find any association between the parameters other than HbA1c and PWV. One reason for this is that the sample size of our study was small and did not have sufficient power.

Supplementary Table S4 shows the proportions of the three treatment methods during the observation period (MDI, CSII, and switching from MDI to CSII, excluding two patients whose treatment was changed from CSII to MDI) in the groups according to the degree of progression of complications (each group in Figure 1). Although the proportion of each treatment method did not seem to differ greatly between most groups, the treatment method of all patients was MDI in Group 1 in Figure 1A. Although the sample size of Group 1 in Figure 1A was small, the progression of eGFR decline was thought to may occur more frequently in patients using MDI. However, the correlation between the outcome variables and treatment methods was not significant in the decision tree analysis. One of the reasons why no correlation was found is thought to be that the sample size of the present study was insufficient. Further studies with larger sample sizes are necessary to clarify the correlation between the progression of diabetic complications and treatment methods. Additionally, the results of the present study suggest that regardless of the treatment method, controlling the factors detected in this study is important for suppressing the progression of diabetic complications.

### 4.3 Limitations

The strengths of our study are that it is the first to evaluate changes in glycemic control and diabetic complications over time in Japanese patients with juvenile‐onset T1D and the factors associated with multiple indicators related to diabetic complications using a decision tree analysis.

However, this study has some limitations. First, this was a retrospective observational study, and the observation period, number of visits, and timing of visits differed among the patients. Small changes in indicators related to diabetic complications may not have been detected in patients with short observation periods. Nineteen of the 129 patients (14.7%) had only two visits; in these patients, the annual changes in eGFR, UAE, CIMT, and baPWV calculated using the linear regression model and the parameters calculated using the trapezoidal rule method might not reflect exact values.

Second, in our health‐check program, other diabetic complications (for instance, such as neuropathy and peripheral artery disease) were not assessed systematically or regularly.

Third, the HbA1c values used in the analysis were only those measured at the time of the annual checkup visit and may not reflect the glycemic control status in patients with large fluctuations in HbA1c throughout the year.

Fourth, we were unable to obtain data from continuous glucose monitoring system or flash glucose monitoring system in this study, and thus, we were unable to evaluate indicators of glycemic excursion such as glucose management indicator, % coefficient of variation, and time in range.

Fifth, seven patients were taking antidiabetic drugs (six patients were taking α‐glucosidase inhibitor and one was taking α‐glucosidase inhibitors and metformin). Due to the small sample size of patients taking antidiabetic drugs, we could not assess the effects of these drugs.

## 5 CONCLUSIONS

A 19‐year follow‐up study of juvenile‐onset T1D in Japanese patients showed improvements in glycemic control and UAE over time, whereas eGFR, CIMT, and baPWV worsened over time. As factors associated with the progression of diabetic complications, eGFR was associated with HbA1c and GA/HbA1c ratio. UAE was associated with HbA1c and SBP levels. CIMT was associated with LDL‐C/HDL‐C ratio, GA/HbA1c ratio, and BMI. baPWV is associated with HbA1c level. This suggests that these parameters are important risk factors for progression of indicators related to diabetic complications in this population.

## AUTHOR CONTRIBUTIONS

All named authors meet the International Committee of Medical Journal Editors (ICMJE) criteria for the authorship of this manuscript and take responsibility for the integrity of the work. All authors contributed to the study design and were involved in all stages of manuscript development. Takafumi Masuda drafted the manuscript. All authors participated in data analysis and interpretation, reviewed and edited the manuscript, and approved the final version. Naoto Katakami is the principal guarantor of this work, has complete access to all data, and takes responsibility for the integrity of the data and the accuracy of the data analysis.

## FUNDING INFORMATION

Financial support for this study, including fees for the assistance and processing of the article by the journal, was provided by the Japan Diabetes Foundation.

## CONFLICT OF INTEREST STATEMENT

Iichiro Shimomura is an editorial board member of the *Journal of Diabetes* and a coauthor of this article. To minimize bias, he was excluded from all editorial decision‐making related to the acceptance of this article for publication. The authors declare no conflicts of interest.

## Supporting information


**Supplementary Table S1.** Annual changes in each parameter evaluated using a linear mixed model for patients with an observation period of two points or more.
**Supplementary Table S2.** Annual change in each parameter evaluated using a linear mixed model for patients with the observation period of 10 points or more.
**Supplementary Table S3.** Associations of glycated hemoglobin (HbA1c), glycoalbumin (GA)/HbA1c ratio, and M‐value with continuous subcutaneous insulin infusion (CSII) use evaluated using a linear mixed model.
**Supplementary Table S4.** Proportion of treatments during the observation period for each group shown in Figure 1.
**Supplementary Figure S1.** Decision tree models for progression of diabetic complications on sensitivity analysis.
