# Supplementary material for: Evaluation of changes in glycemic control and diabetic complications over time and factors associated with the progression of diabetic complications in Japanese patients with juvenile‐onset type 1 diabetes mellitus
Source: J Diabetes. 2023 Oct 18;16(2):e13486. doi: 10.1111/1753-0407.13486 (PMC10859312; doi:10.1111/1753-0407.13486)
Supplement: Supplementary file 1 — Supplementary Table S1. Annual changes in each parameter evaluated using a linear mixed model for patients with an observation period of two points or more. Supplementary Table S2. Annual change in each parameter evaluated using a linear mixed model for patients with the observation period of 10 points or more. Supplementary Table S3. Associations of glycated hemoglobin (HbA1c), glycoalbumin (GA)/HbA1c ratio, and M‐value with continuous subcutaneous insulin infusion (CSII) use evaluated using a linear mixed model. Supplementary Table S4. Proportion of treatments during the observation period for each group shown in Figure 1. Supplementary Figure S1. Decision tree models for progression of diabetic complications on sensitivity analysis. [file JDB-16-e13486-s001.docx]

**Supplementary Table 1** | Annual changes in each parameter evaluated using a linear mixed model for patients with an observation period of two points or more

| Parameters | n | Estimate | P value |
| --- | --- | --- | --- |
| BMI (kg/m^2^/year) | 129 | 0.105 | <0.001 |
| HbA1c (%/year) | 129 | −0.042 | <0.001 |
| GA (%/year) | 127 | −0.727 | <0.001 |
| GA/HbA1c ratio (/year) | 127 | −0.080 | <0.001 |
| LDL-C (mg/dl/year) | 129 | 0.720 | <0.001 |
| Log_10_TG (mg/dl/year) | 129 | −0.001 | 0.329 |
| HDL-C (mg/dl/year) | 129 | 0.131 | 0.005 |
| LDL-C/HDL-C ratio (/year) | 129 | 0.009 | <0.001 |
| non-HDL-C (mg/dl/year) | 129 | 0.736 | <0.001 |
| Systolic blood pressure (mmHg/year) | 129 | 0.258 | <0.001 |
| Diastolic blood pressure (mmHg/year) | 129 | 0.074 | 0.054 |
| M-value (/year) | 93 | −1.447 | 0.040 |
| AUC of glucose (mg/dl h/year) | 93 | −0.476 | 0.080 |
| Severe hypoglycemia (times/year^2^) | 125 | 0.011 | 0.335 |
| eGFR (mL/min/1.73 m^2^/year) | 129 | −1.310 | <0.001 |
| Log_10_UAE (mg/g Cre/year) | 128 | −0.012 | <0.001 |
| CIMT (mm/year) | 129 | 0.027 | <0.001 |
| baPWV (cm/s/year) | 128 | 10.67 | <0.001 |

“Estimate” is the estimated amount of change in each parameter per year.

BMI, body mass index; HbA1c, glycated hemoglobin; GA, glycoalbumin; LDL-C, low-density lipoprotein cholesterol; TG, triglyceride; HDL-C, high-density lipoprotein cholesterol; AUC, area under the curve; M-value, Morbus value; eGFR, estimated glomerular filtration rate; UAE, urinary albumin excretion rate; CIMT, carotid intima–media thickness; baPWV, brachial–ankle pulse wave velocity.

**Supplementary Table 2** | Annual change in each parameter evaluated using a linear mixed model for patients with the observation period of 10 points or more

| Parameters | n | Estimate | P value |
| --- | --- | --- | --- |
| BMI (kg/m^2^/year) | 69 | 0.108 | <0.001 |
| HbA1c (%/year) | 69 | −0.042 | <0.001 |
| GA (%/year) | 69 | −0.754 | <0.001 |
| GA/HbA1c ratio (/year) | 69 | −0.084 | <0.001 |
| LDL-C (mg/dl/year) | 69 | 0.715 | <0.001 |
| Log_10_TG (mg/dl/year) | 69 | −0.0002 | 0.827 |
| HDL-C (mg/dl/year) | 69 | 0.136 | 0.006 |
| LDL-C/HDL-C ratio (/year) | 69 | 0.009 | <0.001 |
| non-HDL-C (mg/dl/year) | 69 | 0.767 | <0.001 |
| Systolic blood pressure (mmHg/year) | 69 | 0.279 | <0.001 |
| Diastolic blood pressure (mmHg/year) | 69 | 0.070 | 0.084 |
| M-value (/year) | 43 | −1.225 | 0.064 |
| AUC of glucose (mg/dl h/year) | 43 | 0.022 | 0.941 |
| Severe hypoglycemia (times/year^2^) | 68 | 0.012 | 0.314 |
| eGFR (mL/min/1.73 m^2^/year) | 69 | −1.279 | <0.001 |
| Log_10_UAE (mg/g Cre/year) | 69 | −0.013 | <0.001 |
| CIMT (mm/year) | 69 | 0.027 | <0.001 |
| baPWV (cm/s/year) | 65 | 10.62 | <0.001 |

“Estimate” is the estimated amount of change in each parameter per year.

BMI, body mass index; HbA1c, glycated hemoglobin; GA, glycoalbumin; LDL-C, low-density lipoprotein cholesterol; TG, triglyceride; HDL-C, high-density lipoprotein cholesterol; AUC, area under the curve; M-value, Morbus value; eGFR, estimated glomerular filtration rate; UAE, urinary albumin excretion rate; CIMT, carotid intima–media thickness; baPWV, brachial–ankle pulse wave velocity.

**Supplementary Table 3** | Associations of HbA1c, GA/HbA1c ratio, and M-value with CSII use evaluated using a linear mixed model

| Parameters | n | Estimate | P value |
| --- | --- | --- | --- |
| HbA1c (%/year) | 129 | −0.560 | <0.001 |
| GA/HbA1c ratio (/year) | 128 | −0.507 | <0.001 |
| M-value (/year) | 117 | −20.94 | 0.012 |

“Estimate” is the estimated amount of change in each parameter by CSII use.

CSII, continuous subcutaneous insulin infusion; HbA1c, glycated hemoglobin; GA, glycoalbumin; M-value, Morbus value.

Supplementary Table 4 | Proportion of treatments during the observation period for each group shown in Figure 1.

| Figure 1 (A) | All Patients  (n=88) | Group 1  (n=9) | Group 2  (n=30) | Group 3  (n=49) |
| --- | --- | --- | --- | --- |
| MDI | 49 (55.7%) | 9 (100.0%) | 15 (50.0%) | 25 (51.0%) |
| CSII | 10 (11.4%) | 0 (0%) | 4 (13.3%) | 6 (12.2%) |
| Switching MDI to CSII | 29 (33.0%) | 0 (0%) | 11 (36.7%) | 18 (36.7%) |

| Figure 1 (B) | All Patients  (n=87) | Group 1  (n=11) | Group 2  (n=9) | Group 3  (n=67) |
| --- | --- | --- | --- | --- |
| MDI | 49 (56.3%) | 7 (63.6%) | 5 (55.6%) | 37 (55.2%) |
| CSII | 13 (14.9%) | 2 (18.2%) | 2 (22.2%) | 9 (13.4%) |
| Switching MDI to CSII | 25 (28.7%) | 2 (18.2%) | 2 (22.2%) | 21 (31.3%) |

| Figure 1 (C) | All Patients  (n=88) | Group 1  (n=23) | Group 2  (n=11) | Group 3  (n=31) | Group 4  (n=23) |
| --- | --- | --- | --- | --- | --- |
| MDI | 48 (54.5%) | 11 (47.8%) | 8 (72.7%) | 16 (51.6%) | 13 (56.5%) |
| CSII | 13 (14.8%) | 1 (4.3%) | 2 (18.2%) | 8 (25.8%) | 2 (8.7%) |
| Switching MDI to CSII | 27 (30.7%) | 11 (47.8%) | 1 (9.1%) | 7 (22.6%) | 8 (34.8%) |

| Figure 1 (D) | All Patients  (n=76) | Group 1  (n=9) | Group 2  (n=67) |
| --- | --- | --- | --- |
| MDI | 36 (47.4%) | 5 (55.6%) | 31 (46.3%) |
| CSII | 12 (15.8%) | 3 (33.3%) | 9 (13.4%) |
| Switching MDI to CSII | 28 (36.8%) | 1 (11.1%) | 27 (40.3%) |

The proportion of treatment in each group of Figure 1 is shown, excluding patients who switched from CSII to MDI during the observation period (2 out of 129 total patients).

Patients with MDI throughout the observation period (MDI), those with CSII throughout the observation period (CSII), and those who switched from MDI to CSII during the observation period (Switching MDI to CSII).

MDI, multiple daily injections; CSII, continuous subcutaneous insulin infusion.

Supplementary Figure 1 | Decision tree models for progression of diabetic complications
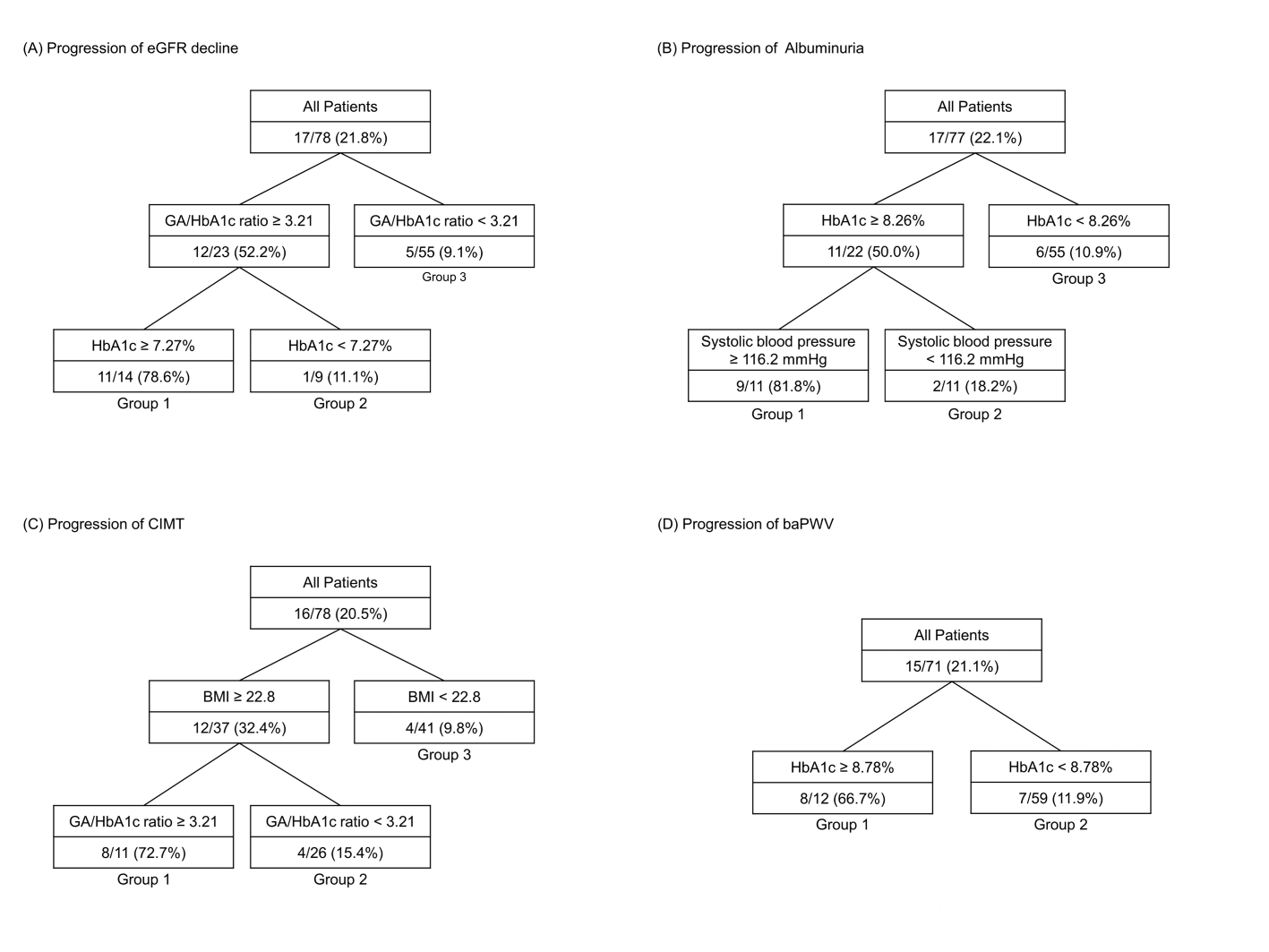
on sensitivity analysis

The upper part of each node represents branching conditions. The lower part of each node shows “n of outcome occurrence/n of patients (% of outcome occurrence).”

(A) Decision tree models for the progression of eGFR decline; (B) decision tree models for the progression of albuminuria; (C) decision tree models for the progression of CIMT; and (D) decision tree models for the progression of baPWV.

Abbreviations: eGFR, estimated glomerular filtration rate; CIMT, carotid intima–media thickness; baPWV, brachial–ankle pulse wave velocity; GA, glycoalbumin; HbA1c, glycated hemoglobin; BMI, body mass index.
